# Bioenergetics of murine lungs infected with respiratory syncytial virus

**DOI:** 10.1186/1743-422X-10-22

**Published:** 2013-01-15

**Authors:** Ahmed R Alsuwaidi, Sheela Benedict, Jose Kochiyil, Farah Mustafa, Stacey M Hartwig, Saeeda Almarzooqi, Alia Albawardi, Tahir A Rizvi, Steven M Varga, Abdul-Kader Souid

**Affiliations:** 1 Departments of Pediatrics, College of Medicine and Health Sciences, UAE University, P.O. Box 17666, Al Ain, United Arab Emirates; 2 Departments of Biochemistry, College of Medicine and Health Sciences, UAE University, P.O. Box 17666, Al Ain, United Arab Emirates; 3 Departments of Pathology, College of Medicine and Health Sciences, UAE University, P.O. Box 17666, Al Ain, United Arab Emirates; 4 Departments of Microbiology, College of Medicine and Health Sciences, UAE University, P.O. Box 17666, Al Ain, United Arab Emirates; 5 Department of Microbiology, University of Iowa, Iowa City, IA, 52242, USA; 6 Department of Pathology, University of Iowa, Iowa City, IA, 52242, USA; 7 Interdisciplinary Graduate Program in Immunology, University of Iowa, Iowa City, IA, 52242, USA

**Keywords:** Respiratory syncytial virus (RSV), Cellular respiration, Oxygen consumption, Cellular ATP, Mitochondria, Caspases

## Abstract

**Background:**

Cellular bioenergetics (cellular respiration and accompanying ATP synthesis) is a highly sensitive biomarker of tissue injury and may be altered following infection. The status of cellular mitochondrial O_2_ consumption of the lung in pulmonary RSV infection is unknown.

**Methods:**

In this study, lung fragments from RSV-infected BALB/c mice were evaluated for cellular O_2_ consumption, ATP content and caspase activity. The disease was induced by intranasal inoculation with the RSV strain A2 and lung specimens were analyzed on days 2–15 after inoculation. A phosphorescence O_2_ analyzer that measured dissolved O_2_ concentration as a function of time was used to monitor respiration. The caspase-3 substrate analogue *N*-acetyl-asp-glu-val-asp-7-amino-4-methylcoumarin (Ac-DEVD-AMC) was used to monitor intracellular caspases.

**Results:**

O_2_ concentration declined linearly with time when measured in a sealed vial containing lung fragment and glucose as a respiratory substrate, revealing its zero-order kinetics. O_2_ consumption was inhibited by cyanide, confirming the oxidation occurred in the respiratory chain. Cellular respiration increased by 1.6-fold (*p*<0.010) and ATP content increased by 3-fold in the first week of RSV infection. Both parameters returned to levels found in uninfected lungs in the second week of RSV infection. Intracellular caspase activity in infected lungs was similar to uninfected lungs throughout the course of disease.

**Conclusions:**

Lung tissue bioenergetics is transiently enhanced in RSV infection. This energy burst, triggered by the virus or virus-induced inflammation, is an early biomarker of the disease and may be targeted for therapy.

## Introduction

Human respiratory syncytial virus (RSV) is the leading cause of bronchiolitis and pneumonia worldwide, infecting nearly all children by 2 years of age [1]. Severe disease occurs in premature infants and individuals with compromised respiratory, cardiac or immune system [2,3]. Furthermore, exposure to RSV early in life may predispose susceptible individuals to asthma [4].

RSV belongs to the *Parmyxoviridae* family of enveloped RNA viruses, specifically the genus *Pneumovirus*[5]. Initially isolated from a chimpanzee, RSV can be found in ruminants, such as sheep, goats and cattle; its main host, however, is humans. The virus has a negative-stranded RNA genome that codes for 10 proteins, including 4 nucleocapsid proteins (N, P, L and M2-1; necessary for RNA replication) and 3 envelope transmembrane proteins (G, F and SH; responsible for virus attachment, membrane fusion, entry and syncytium formation) [5]. The surface fusion protein F and the glycoprotein G serve as the two main targets for antibodies. Variability in the G glycoprotein is primarily responsible for the two clinical strains of RSV, the subgroups A and B [6]. These two subgroups differ mainly in the extracellular domains of G and the small hydrophobic protein SH. Based on sequence variability of the G protein hypervariable region, the subgroups are further divided into clades. The matrix protein (M) is important for viral RNA packaging, while the non-structural proteins have regulatory activities.

RSV infection produces serum and mucosal immune responses that includes IgM, IgA, IgG and IgE; however, the IgG response is short lived and wanes by one year [7]. The primary immune response is not efficient against the initial infection, but results in an effective memory response against reinfection. In addition, the virus generates CD4 and CD8 T cell responses that result in classical interferon-gamma production. Both the humoral and cell-mediated immune responses play roles in virus clearance, but also contribute to the immunopathology of the respiratory tract.

The pathogenesis of RSV is related to its ability to reach the lower respiratory tract, where it can cause bronchiolitis and pneumonia [8]. The RSV strains differ in their virulence, depending upon critical changes in their viral genomes [9]. Overall, subgroup A causes more illness than subgroup B [10]. In the BALB/c mouse model of RSV infection, the virus causes lower respiratory tract infection, making it a good animal model to study [8]. Of note, wild-type inbred mice are described as semi-permissive hosts for human RSV; a very high intranasal inoculum (10^5^ to 10^7^ plaque-forming units per mouse) is usually administered to cause lower respiratory tract disease signs and symptoms such as weight loss, reduced activity, and ruffled fur [11]. Nevertheless, similar to humans, constriction of the airways by mucus production represents a hallmark of disease in mice. Other findings include epithelial hypersensitivity, inflammation, and infiltration by mononuclear cells [9,12]. Furthermore, production of IgE antibodies and skewing of the immune response towards higher Th-2 and lower Th-1 cytokines are thought to contribute to asthma symptoms observed in mice and young infants [7,9].

The molecular pathogenesis of clinically aggressive RSV infection is poorly understood, which partially explains the inability to control the disease at the molecular level [13,14]. For example, information concerning RSV-induced caspases (a series of cysteine, aspartate-specific proteases that mediates apoptosis) in pneumatocytes is inconsistent, and most studies are performed on cell lines. Early studies in the lung epithelial cell line A549 identified apoptotic mediators in response to RSV (e.g., interleukin-1 converting enzyme and CD95), but apoptosis was detected only in ~15% of infected cells [15,16]. Using the same cell line, Bitko and Barik showed RSV-induced apoptosis was mediated by caspase-12 (an endoplasmic reticulum stress response caspase) [17]. Additional *in vitro* data implicated RSV in inducing the anti-apoptotic factor IEX-1L, and that normal signaling through the phosphoinositide 3-kinase pathway blocked RSV-induced apoptosis [17,18]. The study by Kotelkin et al. found transcriptional activations of pro-apoptotic and anti-apoptotic factors in response to RSV in various cell lines [19]. In another study, RSV induced tumor necrosis factor-related apoptosis-inducing ligand (TRAIL) and its receptors and elicited apoptosis associated with activation of caspase-8 (receptor-mediated) and caspase-9 (mitochondrial-associated) [20].

Initiation of apoptosis requires the mitochondria to sense the injury, resulting in leakage of cytochrome c and other small molecular weight pro-apoptotic molecules from the mitochondrial intermembrane space to the cytosol [21]. In the cytosol, cytochrome c binds to the apoptotic protease activating factor-1 (Apaf-1), triggering the caspase cascade. Caspase activation induces mitochondrial perturbations, which involve opening the permeability transition pores and collapsing the electrochemical potential. Thus, induction of apoptosis is linked to mitochondrial dysfunction.

Caspase-3 is also involved in proteolysis of proteins, including poly(ADP ribose) polymerase; it cleaves at the second aspartate in the asp-glu-val-asp sequence. Hence, the synthetic substrate *N*-acetyl-asp-glu-val-asp-7-amino-4-methylcoumarin (Ac-DEVD-AMC) can be used to monitor intracellular caspase-3 activity. The released fluorogenic moiety AMC can be separated on HPLC and detected with a great accuracy [22].

The term “cellular bioenergetics” refers to the biochemical processes involved in energy metabolism (energy conversion or transformation), and the term “cellular respiration” (mitochondrial oxygen consumption) is used to describe the delivery of metabolites and O_2_ to the mitochondria, oxidation of reduced metabolic fuels with the passage of electrons to O_2_, and synthesis of ATP.

Measuring tissue mitochondrial O_2_ consumption, using the principle that O_2_ quenches the phosphorescence of palladium II-*meso*-tetra-(4-sulfonatophenyl)-tetrabenzoporphyrin, has been recently reported [22-25]. This analytical tool allows *in vitro* monitoring of cellular respiration over several hours. Simultaneous determinations of intracellular ATP and caspase activity, however, are necessary, since uncoupling oxidative phosphorylation (accelerated respiration with collapsing cellular ATP) is common after tissue collection. Moreover, caspases are potent inhibitors of the inner mitochondrial membrane function. Therefore, the three parameters (respiration, ATP content and caspase activity) are all necessary for accurate assessment of lung tissue bioenergetics.

The status of lung tissue bioenergetics in RSV infection is currently unknown. It is also unclear whether RSV infection induces pneumatocyte apoptosis and mitochondrial perturbation. Using assays described by us [22-26], these unmet tasks are addressed in this study using a well-established RSV-mouse model system [27].

## Materials and methods

### Reagents

Pd(II) complex of *meso*-tetra-(4-sulfonatophenyl)-tetrabenzoporphyrin **(**Pd phosphor**)** was purchased from Porphyrin Products (Logan, UT). A lyophilized powder of caspase inhibitor I [*N*-benzyloxycarbonyl-val-ala-asp(O-methyl)-fluoromethylketone; zVAD-fmk; *m.w.* = 467.5; pan-caspase inhibitor] was purchased from Calbiochem (La Jolla, CA). Ac-DEVD-AMC (*N*-acetyl-asp-glu-val-asp-7-amino-4-methylcoumarin; *m.w.* = 675.64; caspase-3 substrate) was purchased from Axxora LLC (San Diego, CA). Glucose (anhydrous) and remaining reagents were purchased from Sigma-Aldrich (St. Louis, MO). HEp-2 and Vero cells were obtained from American Type Culture Collection (ATCC; Manassas, VA).

zVAD-fmk (2.14 mM) solution was made by dissolving 1.0 mg in 1.0 mL dimethyl sulfoxide and stored at −20°C. Ac-DEVD-AMC (7.4 mM) solution was made by dissolving 5.0 mg in 1.0 mL dimethyl sulfoxide and stored at −20°C. Phosphate-buffered saline (PBS) with glucose (137 mM NaCl, 2.7 mM KCl, 4.3 mM Na_2_HPO_4_, 1.4 mM KH_2_PO_4_ and 5 mM glucose, *p*H 7.4) was made fresh. Pd phosphor solution (2.5 mg/ml = 2 mM) was prepared in dH_2_O and stored in small aliquots at −20°C. NaCN (1.0 M) was prepared in dH_2_O; the *p*H was adjusted to ~7.0 with 12N HCl and stored at −20°C.

### RSV

RSV strain A2 (RSV-A2) was propagated in the human laryngeal carcinoma cell line, HEp-2 (ATCC). Briefly, cells were grown to ~80% confluence in T-162 flasks and infected with 0.5 ml of ~ 1–2 × 10^7^ plaque-forming units (PFU) per ml with gentle intermittent rocking. Infection was continued at 37°C in a 5% CO_2_ incubator for two days until peeling of monolayer surfaces and formation of syncytia were observed. Cells were scraped from the flask and sonicated on ice until 85 to 95% of the cells had ruptured, releasing virus into the supernatant. The supernatant was collected by centrifugation at 2500 rpm for 15 min at 4°C, distributed into 500 μl aliquots, snap-frozen in liquid nitrogen and stored at −80°C.

### RNA isolation and RT-PCR

RNA was isolated from 20 to 25 mg lung tissue using 1.0 ml TRIZOL reagent (Invitrogen Life Technologies, USA) as per manufacturer’s instruction and stored in ultrapure water at −80°C. For reverse transcription, 5 μg of the extracted lung RNA was DNase-treated with 3 units of RQ1 RNase-free DNase (Promega, Madison, WI) at 37°C for 30 min in the presence of 40 units of Recombinant RNasin (Promega, Madison, WI). The DNased-RNA was RT-PCR amplified with primers for glyceraldehyde 3-phosphate dehydrogenase (GAPDH) to confirm absence of contaminating DNA and converted into cDNA using 300 ng of random hexamers (Metabion, Germany), 400 units of Moloney murine leukemia virus (M-MLV) reverse transcriptase (Promega, Madison WI), and 40 units of RNasin in a 50 μL-reaction volume at 37°C for one hr. One μL of the cDNA was used in a 25 μL reaction volume using 20 μL of RT-PCR Supermix High Fidelity (Invitrogen Life Technologies, USA) with 1 μL of 25 mM MgCl_2_ and 50 ng each of the previously published primers RSVA F and RSVA R [21]. The RT-PCR amplification conditions were as follows: an initial denaturation step at 94°C for 5 min followed by 35 cycles of denaturation at 94°C, annealing at 50°C, and extension at 72°C for 1 min each, and a final extension step at 72°C for 7 min.

### Plaque assay

Viral stocks were assayed for infectivity using a plaque assay on Vero cells (ATCC) as described previously [27]. Briefly, near confluent Vero cells (90 to 95% cultured in 6-well plates) were infected in duplicate with 100 μl of virus stock diluted from 10^-1^ to 10^-9^ in serum-free MEM with intermittent gentle rocking for 90 min. Following infection, the cells were overlaid with 4 ml of a mix of 1% agarose (SeaKem ME agarose; Cambrex) and equal volume of 2x Eagle's Minimal Essential Medium (EMEM; Cambrex). The incubation was continued at 37°C for 5 days before an additional overlay of 2 mL of 1% Seakem ME agarose plus 0.01% neutral red. The plates were left at 25°C to solidify the agarose and then incubated at 37°C for 24 hr. Plaques were counted manually on a light box and the viral titers were expressed as plaque forming units per lung.

### Animals

Male and female BALB/c mice (4 to 10 weeks old, weight ≈18-22 g) used in this study were purchased from the Jackson Laboratory (Bar Harbor, ME). The mice were housed in a room maintained at 22°C with ~60% relative humidity in compliance with NIH guidelines (http://grants.nih.gov/grants/olaw/references/phspol.htm). All mice had *ad libitum* access to standard rodent chow and filtered water. All protocols received approval from the Animal Ethics Committee-UAE University-College of Medicine and Health Sciences. At necropsy, lung specimens were processed for histology, plaque assay, RT-PCR, ATP content, O_2_ consumption and caspase activity.

### Intranasal inoculation

BALB/c mice were anesthetized by sevoflurane inhalation (100 μL per 10 g). The mice were then inoculated intranasally with 100 μl of RSV-A2 (~ 1–2 × 10^6^ PFU) or mock preparation of HEp-2 culture supernatant.

### Lung tissue

Lung specimens were collected on various days after inoculation as previously described [22-24] and *immediately* immersed in ice-cold Krebs-Henseleit (KH) buffer (115 mM NaCl, 25 mM NaHCO_3_, 1.23 mM NaH_2_PO_4_, 1.2 mM Na_2_SO_4_, 5.9 mM KCl, 1.25 mM CaCl_2_, 1.18 mM MgCl_2_, and 6 mM glucose [pH 7.4]) gassed with 95% O_2_: 5% CO_2_. One specimen was immediately transferred to the O_2_ vial for measuring O_2_ consumption. Three specimens were immediately processed for ATP measurements. Two specimens were immediately placed in the caspase reactions (with and without zVAD-fmk). Specimens were also processed for histology, RT-PCR and plaque assay.

For histology, specimens were fixed in 4% phosphate-buffered paraformaldehyde and embedded in paraffin wax blocks. Sections of the fixed lung fragments (5–7 μm thickness) were stained with haematoxylin and eosin and examined under a light microscope. For O_2_ measurements, specimens were placed in 1.0 ml of air-saturated KH buffer containing 0.5% fat-free bovine albumin and 3 μM Pd phosphor. For viral detection, specimens were homogenized in TRIZOL (Invitrogen, USA) and the supernatants stored at −80°C for RNA extraction and RT-PCR.

### Oxygen measurement

Phosphorescence O_2_ analyzer was used to monitor O_2_ consumption by the lung specimens [22-24]. O_2_ detection was performed with the aid of Pd phosphor that had absorption maximum at 625 nm and phosphorescence maximum at 800 nm. Samples were exposed to light flashes (600 per min) from a pulsed light-emitting diode array with peak output at 625 nm (OTL630A-5-10-66-E, Opto Technology, Inc., Wheeling, IL). Emitted phosphorescent light was detected by a Hamamatsu photomultiplier tube (928) after first passing it through a wide-band interference filter centered at 800 nm. The amplified phosphorescence decay was digitized at 1.0 MHz by a 20-MHz A/D converter (Computer Boards, Inc., Mansfield, MA).

A program was developed using Microsoft Visual Basic 6, Microsoft Access Database 2007, and Universal Library components (Universal Library for Measurements Computing Devices; http://www.mccdaq.com/daq-software/universal-library.aspx). It allowed direct reading from the PCI-DAS 4020/12 I/O Board (PCI-DAS 4020/12 I/O Board; http://www.mccdaq.com/pci-data-acquisition/PCI-DAS4020-12.aspx). The pulse detection was accomplished by searching for 10 phosphorescence intensities >1.0 volt (by default). Peak detection was accomplished by searching for the highest 10 data points of a pulse and choosing the data point closest to the pulse decay curve [25].

The phosphorescence decay rate (1/τ) was characterized by a single exponential; I = Ae^-*t*/τ^, where I = Pd phosphor phosphorescence intensity [28]. The values of 1/τ were linear with dissolved O_2_: 1/τ[O_2_, where 1/τ = the phosphorescence decay rate in the presence of O_2_, 1/τ^o^ = the phosphorescence decay rate in the absence of O_2_, and *k*_q_ = the second-order O_2_ quenching rate constant in s^-1^ · μM^-1^.

Lung tissue respiration was measured at 37°C in 1-mL sealed vials. Mixing was with the aid of parylene-coated stirring bars. In vials sealed from air, [O_2_] decreased linearly with time, indicating the kinetics of mitochondrial O_2_ consumption was zero-order. The rate of respiration (*k*, in μM O_2_ min^-1^) was thus the negative of the slope d[O_2_]/d*t*. Sodium cyanide (NaCN) inhibited respiration, confirming O_2_ was being consumed in the mitochondrial respiratory chain.

The calibration reaction contained PBS with 3 μM Pd phosphor, 0.5% fat-free albumin, 50 μg/mL glucose oxidase and various concentrations of β-glucose. The values of 1/τ were linear with [β-glucose]; the value of *k*_q_ was the negative of the slope (*k*_q_ = 101.1 s^-1^ · μM^-1^). The value of 1/τ for air-saturated solution (without glucose) was 28,330 sec^-1^ (coefficient of variation, C_v_ = 12%) and for O_2_-depleted solution (with 500 μM β-glucose, 1/τ_o_) 2,875 s^-1^ (C_v_ = 1%). The high values of C_v_ for the air-saturated solutions were due to the lower phosphorescence intensities with high [O_2_ (little light reaching the photomultiplier tube). [O_2_ was calculated using, 1/τ = 1/τ^o^ + *k*_*q*_[O_2_[28].

### ATP content

Lung tissue fragments were homogenized in 0.5 ml of ice-cold 2% trichloroacetic acid for 2 min. The supernatants were collected by centrifugation (1000x*g* at 4°C for 5 min) and stored at −20°C until analysis. Immediately before ATP measurements, the samples were neutralized with 0.5 ml 100 mM Tris-acetate, 2 mM EDTA (final pH, 7.75). ATP concentration was determined using the Enliten ATP Assay System (Bioluminescence Detection Kit, Promega, Madison, WI). Briefly, 2.5 μl of the supernatant was added to 25 μl of the luciferin/luciferase reagent. The luminescence intensity was measured at 25°C using Glomax Luminometer (Promega, Madison, WI). The standard was linear with [ATP] (10 pM to 100 nM, *R*^*2*^ >0.9999).

### Caspase activity

Lung specimens (~20 mg) were incubated at 37°C in KH buffer continuously gassed with 95% O_2_: 5% CO_2_ with and without 32 μM zVAD-fmk for 10 min. Ac-DEVD-AMC (37 μM) was then added and the incubations continued for additional 20 min (final volume, 1.0 ml). The tissue was disrupted by vigorous homogenization and passages through a 27-G needle. The Ac-DEVD-AMC cleavage reaction was quenched with tissue disruption. The supernatant was collected by centrifugation (16,300 *g* for 90 min) through a Microcentrifuge Filter (nominal molecular weight limit = 10,000 Dalton, Sigma^©^), separated on HPLC, and analyzed for the free fluorogenic AMC moiety.

### HPLC

The analysis was performed on a Waters 1525 reversed-phase HPLC system (Spectra Lab Scientific Inc., Alexandria, VA) that consisted of a manual injector, pump and fluorescence detector. The excitation wavelength used was 380 nm and the emission wavelength 460 nm. Solvents A and B were HPLC-grade CH_3_OH:dH_2_O (1:1; isocratic). The Ultrasphere IP column (4.6 × 250 mm, Beckman) was operated at 25°C at 1.0 ml/min. The run time was 15 min.

### Statistical analysis

Data were analyzed using SPSS statistical package (version 19). The nonparametric test (2 independent variables) Mann–Whitney was used to compare infected and uninfected samples.

## Results

**Figure 1:**
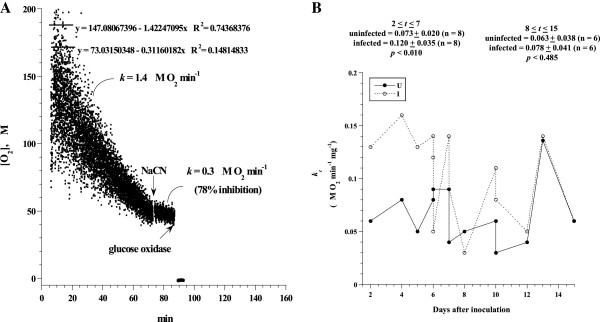
**Lung respiration in RSV-infected and uninfected BALB/c mice.** (**A**) A representative run of O_2_ consumption by lung specimen (26 mg) from infected mouse on day 6. The rate of O_2_ consumption, *k,* was set as the negative of the slope of [O_2_] *vs. t* (1.4 μM O_2_ min^-1^). The value of *k* after the addition of 10 μM NaCN was 0.3 (78% inhibition), confirming the oxidation occurred mainly in the respiratory chain. Glucose oxidase (catalyzes the reaction of D-glucose + O_2_ to D-glucono-δ-lactone + H_2_O_2_) depleted remaining O_2_ in the solution. (**B**) The values of *k*_*c*_ (μM O_2_ min^-1^ mg^-1^) for all experiments are plotted as a function of days after inoculation with RSV strain A2 (infected, I) or mock preparation of HEp-2 culture supernatant (uninfected, U). “n” represents number of mice. The data represent at least 10 independent experiments.

*RSV infection enhances lung tissue bioenergetics in BALB/c mice.* The rates of O_2_ consumption by infected and uninfected lung tissue were determined *immediately* after sample collection. A representative run of lung fragment (26 mg) collected on day 6 after inoculation with RSV-A2 is shown in Figure 1A. The rate of respiration (*k,* the negative of the slope of [O_2_] *vs. t*) was 1.4 μM O_2_ min^-1^. This rate decreased to 0.3 μM O_2_ min^-1^ (78% inhibition) after the addition of 10 mM sodium cyanide (a specific poison of the cytochrome oxidase), confirming the oxidation occurred mainly in the mitochondrial respiratory chain. Glucose oxidase (catalyzes the reaction of D-glucose + O_2_ to D-glucono-δ-lactone + H_2_O_2_) depleted the remaining O_2_ in the solution, confirming the cyanide inhibition of O_2_ consumption occurred despite available dissolved O_2_ in the solution. The corrected rate of respiration (*k*_*c*_) was expressed in μM O_2_ min^-1^ mg^-1^, e.g., in Figure 1A, *k*_*c*_ = 0.05. The values of *k*_*c*_ were plotted in Figure 1B as a function of days after inoculation with RSV- A2 or mock preparation of HEp-2 culture supernatant. The *k*_*c*_ values for 2 ≤ *t* ≤ 7 days after inoculation with mock were 0.073 ± 0.020 (n = 8) and with RSV were 0.120 ± 0.035 (n = 8), *p* < 0.010. For 8 ≤ *t* ≤ 15 days, *k*_*c*_ for inoculation with mock was 0.063 ± 0.038 (n = 6) and with RSV was 0.078 ± 0.041 (n = 6), *p* < 0.485. Thus, lung tissue respiration was enhanced by about 65% in the first week of RSV infection.

**Figure 2:**
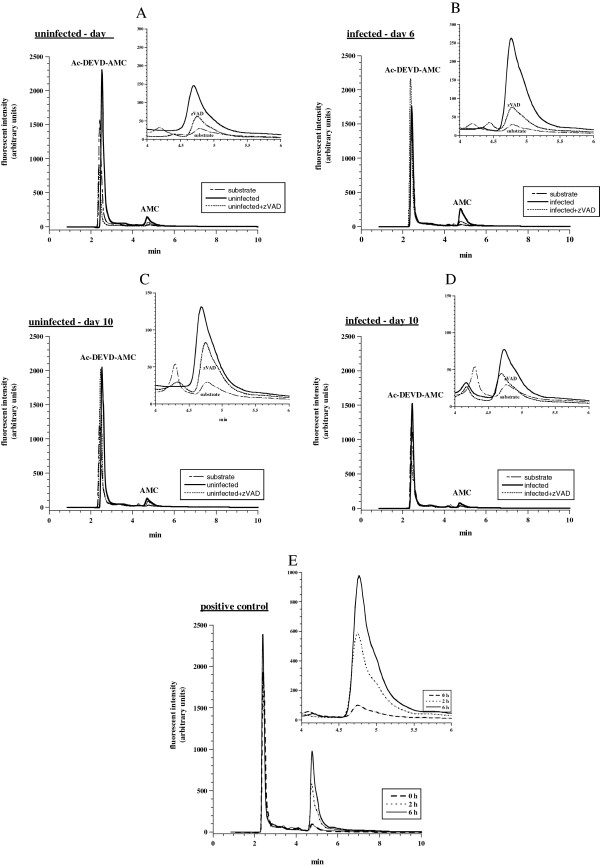
**Lung caspase activity in RSV-infected and uninfected mice.** Representative HPLC runs of the Ac-DEVD cleavage reaction by lung specimens from infected (**B** and **D**) and uninfected (**A** and **C**) mice on days 6 (**A-B**) and 10 (**C-D**) after inoculation are shown. Briefly, BALB/c mice were inoculated on day 0 with the RSV-A2 or the mock preparation of HEp-2 culture supernatant. Lung specimens were collected on days 6 and 10 after inoculation and immediately incubated at 37°C in 1.0 mL KH buffer (continuously gassed with 95% O_2_: 5% CO_2_) with and without 32 μM zVAD-fmk (pan-caspase inhibitor) for 10 min. Ac-DEVD-AMC (37 μM) was then added and the incubations continued for additional 20 min. The tissues were disrupted by vigorous homogenization and the supernatants were separated on HPLC and analyzed for the AMC peak (retention time, *R*_*t*_, ~4.8 min; the AMC moiety reflects caspase-3 activity). Ac-DEVD-AMC had a *R*_*t*_ of ~2.5 min. The substrate runs were without lung specimen and showed negligible AMC peak areas. Panel E (positive control) shows increased lung tissue caspase activity in a sample from uninfected mouse incubated *in vitro* in KH buffer (without oxygenation) for 0, 2 and 6 h. The AMC peak area (arbitrary units mg^-1^) at 0 h (immediately post tissue collection) was 482,115, at 2 h was 3,417,616 and at 6 h was 6,456,028. Similar results were noted in 10 independent experiments spanning the course of the disease over 15 days.

*Intracellular lung caspase activity is not altered with RSV infection of BALB/c mice.* We next sought to determine whether RSV-A2 infection activates intracellular caspases in the lung tissue. Figure 2 shows representative HPLC runs of the Ac-DEVD cleavage reaction by lung specimens from infected and uninfected mice on days 6 and 10 after inoculation. Briefly, BALB/c mice were inoculated on day 0 with the RSV-A2 or mock preparation of HEp-2 culture supernatant. Lung specimens were then incubated at 37°C in 1.0 ml KH buffer (continuously gassed with 95% O_2_: 5% CO_2_) with and without 32 μM zVAD-fmk (a pan-caspase inhibitor) for 10 min. Ac-DEVD-AMC (37 μM) was added and the incubations continued for additional 20 min. The tissues were disrupted by vigorous homogenization and the supernatants were separated on HPLC and analyzed for the AMC peak (retention time, *R*_*t*_, ~4.8 min). The substrate runs were exactly as above, but without lung tissue; AMC peak areas for the substrate runs were negligible (Figure 2A-D). For uninfected lungs on day 6 (Figure 2A), the AMC peak area (arbitrary units ÷ 10^6^ ÷ specimen weight in mg) without zVAD-fmk was 0.62 and with zVAD-fmk 0.44 (29% inhibition). The corresponding values for infected lungs were 1.17 (a 1.9-fold increase) and 0.36 (69% inhibition), respectively (Figure 2B). For uninfected lungs on day 10 (Figure 2C), the AMC peak area without zVAD-fmk was 0.81 and with zVAD-fmk 0.26 (67% inhibition). The corresponding values for infected lungs were 0.26 and 0.23 (12% inhibition), respectively (Figure 2D). Thus, the AMC moieties released in the presence of zVAD-fmk were negligible (see panel E) and reflected mostly non-specific hydrolysis of Ac-DEVD-AMC. Otherwise, the sensitivity of the cleavage reaction to zVAD-fmk typically exceeds 80%, confirming the substrate was mainly hydrolyzed by caspases.

A positive control for increased lung tissue caspase activity is shown in Figure 2E. Lung sample was collected from an uninfected mouse and incubated *in vitro* in KH buffer (without gassing with 95% O_2_: 5% CO_2_) for 0, 2 and 6 h. Under this condition, intracellular caspases are typically induced by 2 h (personal observation). The AMC peak area (arbitrary units ÷ 10^6^ ÷ specimen weight in mg) at 0 h (immediately post tissue collection) was 0.5, at 2 h was 3.4 and at 6 h was 6.5. Thus, the amounts of AMC moieties shown in panels A-D for infected and uninfected lungs were relatively negligible. The same results were confirmed in 10 independent experiments spanning the course of the disease over 12 to 15 days as shown in the examples below.

**Figure 3:**
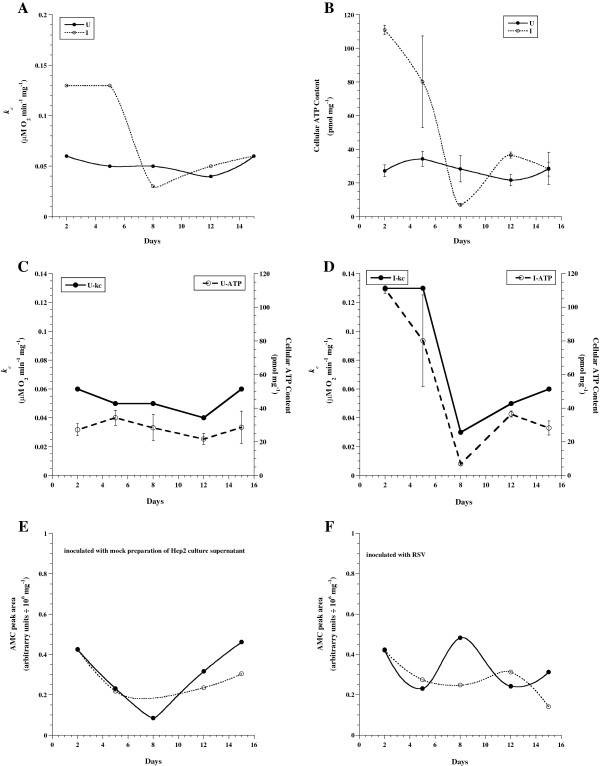
**Representative experiment of lung respiration, ATP content and caspase activity in RSV-infected and uninfected mice on days 2, 5, 8, 12 and 15 after inoculation.** The values *k*_*c*_ (**A**) and ATP (**B**) are plotted as a function of days after inoculation with RSV-A2 (infected, I) or mock preparation of HEp-2 culture supernatant (uninfected, U). The values of *kc* and ATP are re-plotted for uninfected (**C**) and infected (**D**) mice to further illustrate coupling of cellular respiration with ATP content. Panels E-F shows AMC peak area (reflects caspase-3 activity, done in duplicates) in uninfected (**E**) and infected (**F**) mice (solid circles are without zVAD-fmk and open circles are with zVAD-fmk).

*Kinetics of lung bioenergetics and caspase activity in BALB/c mice infected with RSV-A2.* Figure 3 (Panels A-F) shows a representative experiment of lung respiration, ATP content and caspase activity in infected and uninfected mice on days 2, 5, 8, 12 and 15 after inoculation. The values of *k*_*c*_ (Panels A, C and D), ATP (in triplicates, Panels B, C and D) and AMC peak area (in duplicates, Panels E and F) were plotted as a function of days after inoculation with RSV-A2 or mock preparation of HEp-2 culture supernatant.

In infected lungs, rates of respiration and ATP contents were higher on days 2 and 5 than days 8, 12 and 15 (Figure 3, Panels A, B and D). By contrast, the values of *k*_*c*_ and ATP in uninfected lungs were stable from days 2 to 15 (Figure 3C). The relatively low values of *k*_*c*_ and ATP on day 8 were confirmed in an independent experiment, showing *k*_*c*_ = 0.08 μM O_2_ min^-1^ mg^-1^ for uninfected lung and *k*_*c*_ = 0.06 μM O_2_ min^-1^ mg^-1^ for infected lung. Of note, the value of *k*_*c*_ was similar in infected and uninfected lungs on day 1 after inoculation (0.11 and 0.10, respectively). The AMC peak areas on days 2, 5, 8, 12 and 15 after inoculation with RSV-A2 (Figure 3F) were similar to those after inoculation with mock preparation of HEp-2 culture supernatant (Figure 3E).

Similar results were observed in another experiment that measured lung respiration, ATP content and caspase activity in infected and uninfected lungs on days 3, 5, 7, and 10 after inoculation (data not shown). For days 3, 5 and 7 after inoculation, the values of *k*_*c*_ in uninfected lungs were 0.057 ± 0.020 μM O_2_ min^-1^ mg^-1^ and in infected lungs 0.107 ± 0.035 μM O_2_ min^-1^ mg^-1^. On day 10, the value of *k*_*c*_ was 0.090 μM O_2_ min^-1^ mg^-1^ in both infected and uninfected lungs. Similarly, ATP contents in infected lungs were higher than uninfected lungs and caspase activity was negligible.

**Figure 4:**
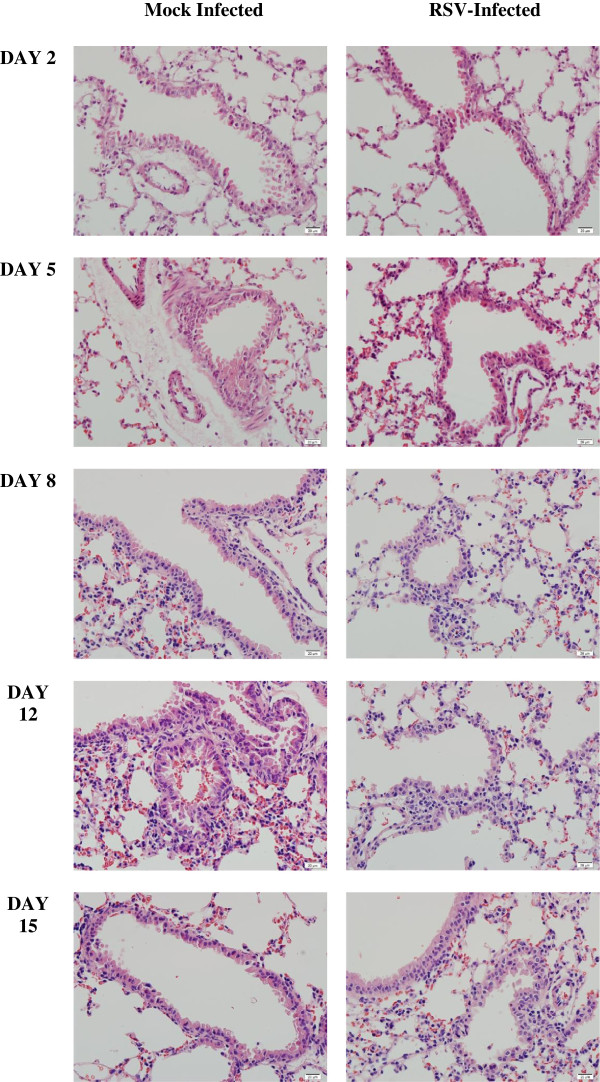
**Lung histology.** Histological sections of mock infected and RSV-infected BALB/c mice on days 2–15 demonstrate lack of peribronchial inflammation on days 2 and 5. There was a mild peribronchiolar and peribronchial inflammation present on days 8, 12 and 15. (Hematoxylin & eosin, 40x).

Histological sections from experiments on days 2–15 demonstrated a lack of peribronchial inflammation on days 2 and 5 (Figure 4). There was a mild peribronchial inflammation present on days 8, 12 and 15 (Figure 4).

**Figure 5:**
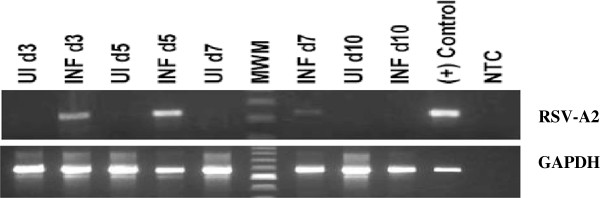
**Reverse transcriptase PCR (RT-PCR).** Whole cell RNA from infected lung tissue on days 3, 5, 7 and 10 after inoculation was isolated, DNase-treated, and converted into cDNA using the reverse transcriptase enzyme. The resulting cDNA was amplified using RSV-A2 specific primers. The distinct band of correct size (334 bp, with intensity peaking on day 5) is absent in the uninfected lung, but present in infected lung and in the cDNA sample from purified RSV virions. The same results were obtained in 2 independent experiments.

*Detection of RSV-A2 in infected mice lungs.* The presence of RSV in infected lungs was verified using reverse transcriptase PCR (RT-PCR) (Figure 5). Whole cell RNA from infected lung tissue was isolated on days 3, 5, 7 and 10 after inoculation, DNase-treated, and converted into cDNA using the reverse transcriptase enzyme. The resulting cDNAs were amplified using RSV-A2 specific primers [29]. The starting RNA was free from contaminating DNA, as evidenced by the lack of amplification of the housekeeping gene *glyceraldehyde 3-phosphate dehydrogenase (GAPDH)* (data not shown). A distinct band of the correct size (334 bp), with intensity peaking on day 5, could be visualized that was absent in the uninfected lung, but present in the cDNA sample from purified RSV virions. This observation confirmed the lung samples were infected with RSV.

Further confirmation of the presence of infectious virus was revealed by the plaque assay [30]. Whole lung titers (tested on days 2, 5, 8 and 10) showed a peak on day 5 with 3 × 10^2^ PFU/lung, while day 8 showed a titer of 2 × 10^1^ PFU/lung. The titers for days 2 and 10 remained below the sensitivity of the assay. These results confirmed the RT-PCR results that the infection peaked on day 5. Similar kinetics of infection has been observed with RSV patient isolates tested in BALB/c mice, where the viral load in the lung peaked on day 4 post-intranasal inoculation of 6–8 week old mice using 10^5^ PFU [9].

## Discussion

The studied murine model shows a transient enhancement of lung tissue bioenergetics (increased cellular mitochondrial O_2_ consumption and ATP content) in the first week of RSV infection (Figure 1 and 3). As previously shown, reproduction of viral particles is highly dependent on host energy metabolism and metabolic fuels are essential for viral survival [31]. Thus, the data suggest that increased pneumatocyte energy conversion (ATP production) is required early in RSV infection to sustain active viral replication.

As shown in Figure 3, cellular ATP in infected lung tissues increased by ~3-fold on days 2 to 5 after inoculation. This finding is consistent with the study of Davis et al. showing a 2-fold increase of nucleotide levels (ATP and UTP) in bronchoalveolar lavage fluid of RSV infected BALB/c mice on day 2. Moreover, inhibition of *de novo* pyrimidine synthesis with leflunomide resulted in reduction of UTP and ATP in the bronchoalveolar lavage fluid, which reversed impaired alveolar fluid clearance, edema and hypoxemia. Thus, understanding the metabolic energy flow may be useful in combating RSV infection [32].

Consistently, hepatitis C virus was found to utilize host lipid metabolism for its survival in a way that promotes liver injuries [33]. Moreover, anti-HCV therapies directed specifically at host metabolic pathways have been successfully developed [34], which highlights the therapeutic potentials of targeting host metabolic pathways to halt viral replication.

Cellular mitochondrial O_2_ consumption and ATP synthesis are linked to the entire metabolism. Therefore, these biomarkers are highly sensitive for detecting changes in cellular energy processes. Both parameters are used here to show increased energy expenditure (requirement) in RSV infected lungs.

The virus or its associated cytokines could drive the observed high-energy demand early in the course of RSV infection. Since viruses typically utilize host resources, it is likely that RSV transforms pneumatocyte metabolism favoring viral survival. The RSV load dynamics, measured by plaque assay in BAL samples of mice, was investigated in one study. RSV load peaked on days 3 to 5 after inoculation (representing active viral replication) and was not detectable on day 7 and thereafter [35]. These results suggest that a high-energy requirement is needed during RSV load peak in the first week of infection. Thus, the energy profiles shown in Figure 3 coincide with the RSV replication and returns to normal state after viral replication ceases. Of note, lung tissue TNF-α Peribronchiolar and peribronchial and INF-γ levels both peaked on day 5 (data not shown).

Peribronchiolar and peribronchial infiltrates were noticeable only on days 8, 12 and 15 after inoculation (Figure 4); that is, after the energy burst. Enhanced (about 40%) cellular mitochondrial oxygen consumption rate was also noted in cultured HEp-2 cells 90 min after the addition of RSV strain A2 (data not shown).

In this model, caspase activity was not noted in the lung tissue during the studied course of RSV infection (Figure 2). This finding correlates with the mild disease observed histologically (Figure 4). Virus strain, inoculation dose and host factors (BALB/c mice are semi-permissive to human RSV infection) are potential contributors to the observed benign disease. It remains to be seen, however, if manipulating these variables will result in infection with increased caspase activity. Alternatively, caspase induction may require a co-infection with bacteria. Substantial caspase activity will impair mitochondrial function and deplete cellular ATP, an event that inevitably leads to cell death. This fact reflects the dependency of mammalian energy supply on aerobic metabolism. Therefore, absence of apoptosis and intact pneumatocyte bioenergetics are favorable prognostic biomarkers.

It is unclear how cellular respiration is accelerated in RSV infected lungs. It is also unknown whether these results are specific for RSV or can be seen with other viruses. Of note, this mechanism could not be investigated *in vitro* since the rate of respiration increased about 4-fold in samples incubated *in vitro* for ≤4 h (Additional file 1: Table S1). This finding most likely reflected uncoupling oxidative phosphorylation as evidenced by the sharp decline in cellular ATP *in vitro* (data not shown).

Nevertheless, the mechanism of accelerated respiration in infected lung was further investigated as follows. First, cellular ATP was also increased in infected lung indicating that the increased energy expenditure was not due to uncoupling oxidative phosphorylation. Second, in glucose-free media, cellular respiration was halted sooner in infected lungs indicating more rapid depletion of the endogenous metabolic fuels in RSV infected lungs. In glucose-free media, the value of *k*_*c*_ for uninfected lung tissue was 0.05 μM O_2_ min^-1^ mg^-1^ and remained the same after the addition of 10 mM glucose at *t* = 50 min. The corresponding values for the RSV infected lung were 0.05 and 0.13, respectively. Therefore, the accelerated respiration in RSV infected was dependent on exogenous glucose as a respiratory substrate.

It is still unclear how the RSV infection could regulate lung tissue cellular energy conversion. Potential mechanisms may include increasing delivery of metabolic fuels to host pneumatocyte, up regulating metabolic enzymes, and preventing inhibitory (regulatory) steps in energy pathways. As noted above for hepatitis C, it remains to be seen whether these biomarkers can be targeted for therapy.

## Competing interests

The authors declare no competing interests.

## Authors’ contributions

ARA, SMV and AKS designed the study, carried out the analysis, interpreted the data and drafted the manuscript. SB measured cellular respiration, ATP content and caspase activity. JK performed the intranasal inoculation. FM, SMH and TR contributed to the viral preparation, plaque assay and RT-PCR. SA and AA examined the histological staining. All authors read and approved the final manuscript.

## Supplementary Material

Additional file 1: Table S1 BALB/c mouse lung respiration on Days 2-15 after inoculation with RSV strain A2 or mock preparation of HEp2 culture supernatant. Respiration was measured immediately after tissue collection (t = 0) and after *in vitro* incubation at 37^o^C in KH buffer (gassed with 95% O_2_: 5% CO_2_) for 2 to 4 h. The increments in the rate of respiration after *in vitro* incubation were due to uncoupling oxidative phosphorylation. Click here for file
