# Supplementary material for: Bioenergetics of murine lungs infected with respiratory syncytial virus
Source: Virol J. 2013 Jan 15;10:22. doi: 10.1186/1743-422X-10-22 (PMC3616819; doi:10.1186/1743-422X-10-22)
Supplement: Additional file 1: Table S1 — BALB/c mouse lung respiration on Days 2-15 after inoculation with RSV strain A2 or mock preparation of HEp2 culture supernatant. Respiration was measured immediately after tissue collection (t = 0) and after in vitro incubation at 37oC in KH buffer (gassed with 95% O2: 5% CO2) for 2 to 4 h. The increments in the rate of respiration after in vitro incubation were due to uncoupling oxidative phosphorylation. [file 1743-422X-10-22-S1.docx]

**Table (Supplement).** **BALB/c mouse lung respiration on Days 2-15 after inoculation with RSV strain A2 or mock preparation of Hep2 culture supernatant.** Respiration was measured immediately after tissue collection (t = 0) and after *in vitro* incubation at 37^o^C in KH buffer (gassed with 95% O_2_: 5% CO_2_) for 2 to 4 h. The increments in the rate of respiration after *in vitro* incubation were due to uncoupling oxidative phosphorylation.

|  |  | ***k_c_***  **(μM O_2_ min^-1^ mg^-1^)** | |
| --- | --- | --- | --- |
|  |  | ***t* = 0 h** | ***t* = 2-4 h** |
| **Day 2** | uninfected | 0.06 | 0.17  0.26 |
|  | infected | 0.13 | 0.18  0.24 |
| **Day 5** | uninfected | 0.05 | 0.23  0.22 |
|  | infected | 0.13 | 0.18  0.26 |
| **Day 8** | uninfected | 0.05 | 0.19  0.16 |
|  | infected | 0.03 | 0.15  0.12 |
| **Day 12** | uninfected | 0.04 | 0.15  0.28 |
|  | infected | 0.05 | 0.19  0.17 |
| **Day 15** | uninfected | 0.06 | 0.19  0.21 |
|  | infected | 0.06 | 0.21  0.22 |
